# Role of Donor-Specific Regulatory T Cells in Long-Term Acceptance of Rat Hind Limb Allograft

**DOI:** 10.1371/journal.pone.0043825

**Published:** 2012-08-29

**Authors:** Yaojun Wang, Zhao Zheng, Yunchuan Wang, Jiaqi Liu, Na Li, Xiaolong Hu, Fu Han, Yang Liu, Dahai Hu

**Affiliations:** Department of Burns and Cutaneous Surgery, Xijing Hospital, Fourth Military Medical University, Xi'an, Shaanxi Province, China; Children's Hospital Boston/Harvard Medical School, United States of America

## Abstract

**Background:**

Vascularized bone marrow transplantation (VBMT) is widely accepted as an efficient means of establishing chimerism and inducing tolerance. However, the mechanism underlying is poorly understood. Recently, regulatory T cells (Tregs) have been shown to play an important role in regulating immune responses to allogeneic antigens. In this study, we explored the role of Tregs in the induction of tolerance in an allogeneic hind limb transplantation model.

**Methodology/Principal Findings:**

Forty-eight Lewis rats were divided into 6 groups. They received isografts and allografts from Brown-Norway hind limbs. Recipients in groups 1 and 2 received isografts and those in the other groups received allografts. The bone components of donor limbs were kept intact in groups 1, 3, and 5 but removed before transplantation into groups 2, 4, and 6. Tapered cyclosporin A (CsA) was administered to recipients in groups 5 and 6 after transplantation. During the 100-day observation period, all isografts survived, but the allografts in groups 3 and 4 were rejected within 8 to 12 days. CsA-treated intact allografts survived rejection-free for more than 100 days, and CsA-treated allografts lacking bone elements were rejected within 2 months. Stable peripheral chimerism and myeloid chimerism were observed in group 5. Declining peripheral chimerism and a lack of myeloid chimerism were observed in group 6. Donor-specific Tregs were exclusively detected in both peripheral blood and in the spleens of long-term recipient rats in group 5, with an increased FoxP3 mRNA expression in the allografts. This was further demonstrated to be responsible for donor-specific hyporeactivity by *in vitro* one-way mixed lymphocyte reaction (MLR).

**Conclusion/Significance:**

Bone components in the allogeneic hind limbs can induce myeloid chimerism and donor-specific Tregs may be essential to tolerance induction. The bone-removal hind limb model may be a suitable counterpart to the induction of tolerance in the study of limb transplantation.

## Introduction

The number of patients undergoing composite tissue defects has increased sharply over the past several years. As of 2005, a total of 1.6 million people are living with the loss of a limb in the U.S. alone, and this number may reach 3.6 million by the year 2050 [1]. People with these defects usually face a number of psychological and social problems, such as social anxiety, lowered self-confidence, negative self-image, depression, and even suicide [2]–[4]. With the use of immunosuppressants, highly developed composite tissue allotransplantation (CTA) techniques make reconstruction possible. However, the long-term survival of the composite tissue allografts (CTAs) is limited due to both chronic rejection and to the side effects associated with immunosuppressants [5].

The hematopoietic chimerism established by bone marrow cell transplantation leads to condition known as tolerance, in which the recipient's immune system is fundamentally reprogrammed and accepts the donor tissue for long periods without rejection [6]–[9]. It has been suggested that vascularized bone marrow transplantation (VBMT) may be superior to conventional bone marrow cell transplantation for the induction of tolerance. This phenomenon is thought to be associated with the bone components, which serve as a vascularized method of delivering donor-origin stem and progenitor cells [10], [11]. However, the exact mechanism remains poorly understood.

Regulatory T cells (Tregs) are a subset of CD4+ T cells. They express FoxP3, a forkhead/winged helix transcription factor, which is important in the regulation of both Treg development and function. Tregs have been found to be effective in the suppression of autoimmunity and alloimmunity [12]. Recently, they have been approved for peripheral tolerance maintenance and long-term graft acceptance [13], [14].

Allogeneic hind limb transplantation, a form of CTA involving different tissues, such as skin, muscle, blood vessels, nerves, and bone, is believed to be more immunogenic than other sets of tissues and that it therefore requires intense immunosuppression [15]. However, the fact that the majority of recipients maintain their transplants on immunosuppression regimens similar to those used in solid organ transplantation suggests that this may not be true [16]–[18]. We speculate that bone components within the allogeneic limbs may be responsible for this. To assess this speculation and explore the mechanism, we utilized a modified hind limb transplantation model to investigate the role of donor bone components in host immunologic responses. We assessed their contributions to the development of chimerism and allograft survival. Given their known activity on tolerance maintenance, the role of FoxP3^+^ Tregs in this model was also investigated. We found that the inclusion of bone components promoted stable myeloid chimerism and prolonged allograft survival was achieved. Donor-specific Tregs were found to be associated with long-term allograft survival, as confirmed by *in vitro* one-way mixed lymphocyte reaction (MLR).

## Results

### Results of the operation

**Figure 1:**
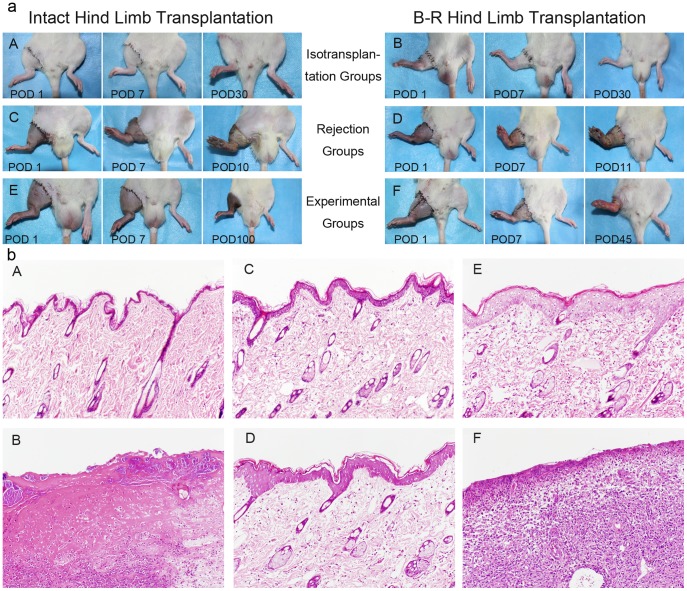
Evaluation of tissue viability and pathological changes of transplanted grafts. (a-A, B): Isografts in groups 1 and 2 survived well. (a-C, D): Allografts in groups 3 and 4 that received N.S showed obvious rejection profiles at 10–12 days postoperatively. (a-E): Allografts in group 5 survived rejection-freely for up to 100 days. With the same CsA protocol, the B–R hind limbs in group 6 were all rejected within 2 months (a-F). (b-A): Skin histologic sections from isograft groups showed normal epidermis and dermis. (b-B): Skin histologic sections from rejection control groups showed necrotic epidermis features of grade 3 rejection reaction at POD 11. (b-C, D): Skin biopsies from CsA treatment groups 5 and 6 at POD 30 showed grade 1 rejection reaction with focal epidermal mononuclear cell infiltration, respectively. (b-E): At POD 100, skin biopsies from recipients in group 5 showed grade 1 rejection and a mild increase in the number of lymphocytes infiltrating the upper dermis at POD 100. (b-F): Skin biopsy from group 6 when the allograft was rejected showed necrotic features of grade 3 rejection reaction in the epidermis and a marked increase in the number of lymphocytes infiltrating all tissues from the dermis down to the muscles (H&E, ×20).

The mean operation time was 150 min in groups 1, 3, and 5 and 180 min in groups 2, 4, and 6. Both groups showed a mean ischemia time of 35 min. No signs of graft versus host disease (GVHD) were observed in all recipient rats during the 100-day observation period. Isografts in groups 1 and 2 survived without signs of rejection (Figs. 1a-A and B). All allografts in groups 3 and 4 showed typical signs of rejection at postoperative days (POD) 4–6. Signs of rejection included bulla formation followed by exudation, epidermolysis, crust formation, and stiffness of limb tissues. Allografts were completely rejected during POD 8–12 (Figs. 1a-C and D). Allografts in group 5 survived rejection-free during the entire observation period (Figs. 1a-E). All bone-removal (B–R) allografts in group 6 showed signs of rejection, including loss of hair, ulceration, epidermolysis, exudation, and stiffness at POD 38–42. Allografts were completely rejected at POD 46–51 (Fig. 1a-F).

### Quantification of hind limb graft perfusion

**Figure 2:**
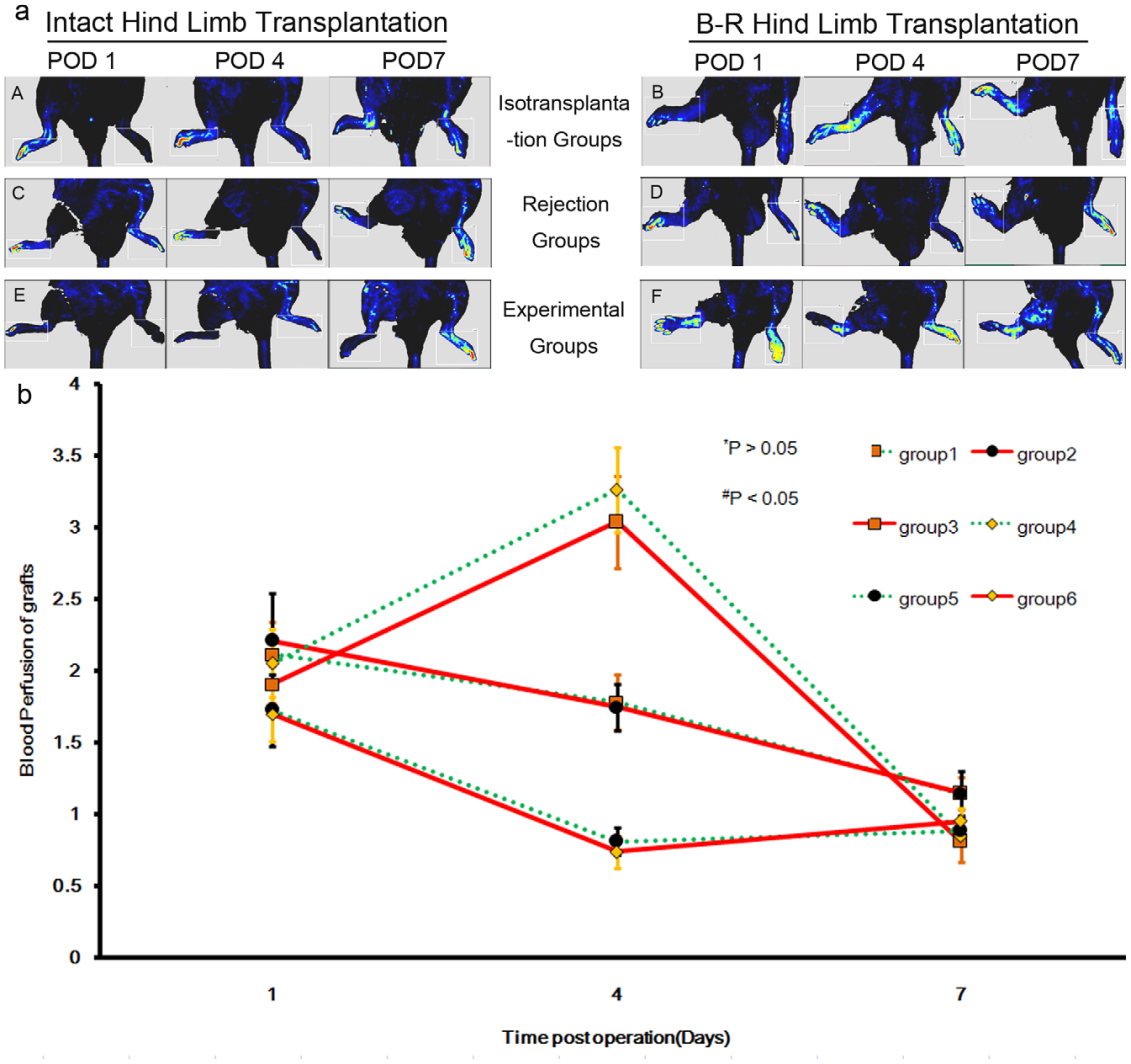
Dynamic changes of blood perfusion in grafts of the recipients. (a) All transplantations were successful, as confirmed by the considerable blood supply of the transplanted grafts according indicated by LDF. (b) The perfusion ratio between intact hind limbs and B–R hind limbs in each group was analyzed. Blood perfusion in isograft controls showed a smooth course of recovery during the observation period. In rejection groups, it peaked at POD 4 and then decreased sharply to a level below that of the contralateral limb. Blood perfusion became undetectable when they were rejected at POD 11. The blood perfusion of transplanted grafts in experimental groups remained low at all times, and ebbed at POD 4. Then it increased to a level below that of the contralateral limb at POD 7. Mean blood perfusion of grafts did not show any significant differences between any two groups given the same treatment at any point in time point (^*^ *P*>0.05). In contrast, all pairs of rejection and experimental groups showed statistical differences at POD 4 (^#^ *P*<0.05).

All grafts showed considerable blood supply during the first 7 days after operation (Fig. 2a). In groups 1 and 2, the blood perfusion in the transplanted grafts were assessed at 2.12±0.22 versus 2.21±0.33 at POD 1 and 1.78±0.19 versus 1.75±0.16 at POD 4, respectively. When it came to POD 7, the perfusion value paralleled with the contralateral hind limb with a perfusion ratio 1.14±0.11 versus 1.14±0.15. Groups 1 and 2 showed no significant differences in perfusion (^*^ *P*>0.05). In groups 3 and 4, the mean blood perfusion of the allografts at POD 1 were 1.9±0.13 versus 2.1±0.24. These values increased more than 3 fold at POD 4 (3.04±0.32 versus 3.27±0.3) and decreased sharply thereafter (0.81±0.14 versus 0.85±0.03). No significant differences were observed between groups 3 and 4 (^*^ *P*>0.05). In contrast, allograft blood perfusion in groups 5 and 6 remained low at all points in time, with the most marked decrease occurring at POD 4 (0.81±0.10 versus 0.74±0.11) and a tendency toward increase at POD 7 (0.88±0.09 versus 0.95±0.12). There were no significant differences between these two groups (^*^ *P*>0.05). However, the rejection and experimental groups showed statistically significant differences in blood perfusion at POD 4 (^#^ *P*<0.05) (Fig. 2b).

### Histopathological evaluation

In the isograft groups (groups 1 and 2), skin biopsy at POD 30 and 100 showed normal epidermis and dermis (Fig. 1b-A). In contrast, allograft skin samples from recipients in groups 3 and 4 showed grade 3 rejection (Fig. 1b-B). Skin biopsy from transplanted allografts in groups 5 and 6 showed grade 1 rejection characterized by focal epidermal mononuclear cell infiltration at POD 30 (Figs. 1b-C, 1D). A mild increase in the number of lymphocytic infiltrates in the upper dermis of POD 100 tissue sections from group 5 was observed (Fig. 1 b-E). In group 6, signs of grade 3 rejection were confirmed by microscopic evaluation in all allografts on the day of rejection (Fig. 1 b-F).

### Donor-specific chimerism in the peripheral blood of recipients

**Figure 3:**
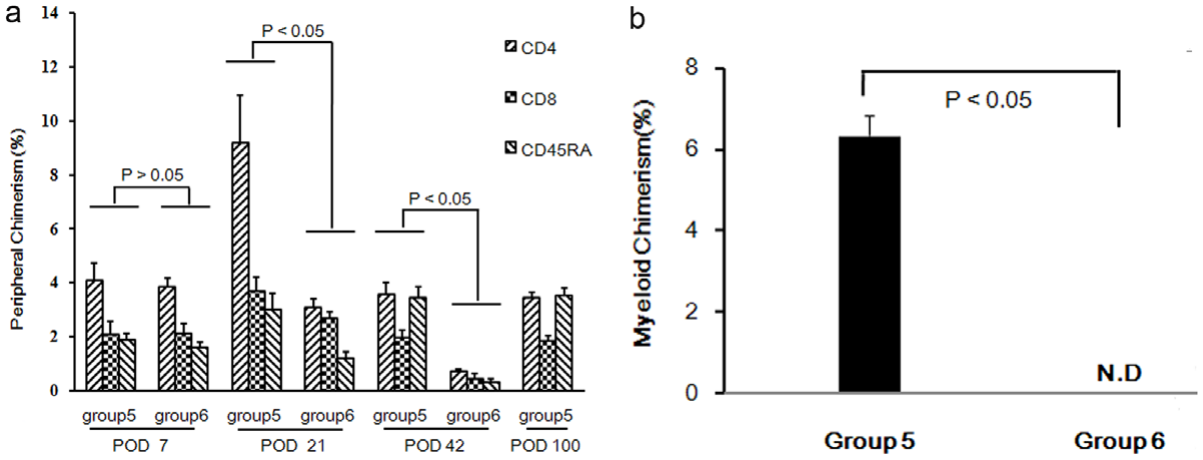
Dynamic changes of peripheral chimerism. Flow cytometry analysis for the presence of donor-specific CD4+/CD8+ T lymphocytes and CD45RA+ B lymphocytes at POD 7, POD 21, and POD 42 of one representative intact allogeneic hind limb recipient with CsA treatment. Levels of donor-specific chimerism were 3.3% in CD4/RT1^n^ cells, 1.2% in CD8/RT1^n^ cells, and 1.6% in CD45RA/RT1^n^ cells at POD 7. They rose to the highest level for donor-origin CD4/RT1^n^ 13.2%, CD8/RT1^n^ 2.9%, and CD45RA/RT1^n^ 2.1% at POD 21, remained at 3.6% CD4/RT1^n^, 2.0% CD8/RT1^n^, and 3.0% CD45RA/RT1^n^. The background levels observed in isograft controls were assessed at 0.3%, 0.2%, and 0.3%, respectively.

**Figure 4:**
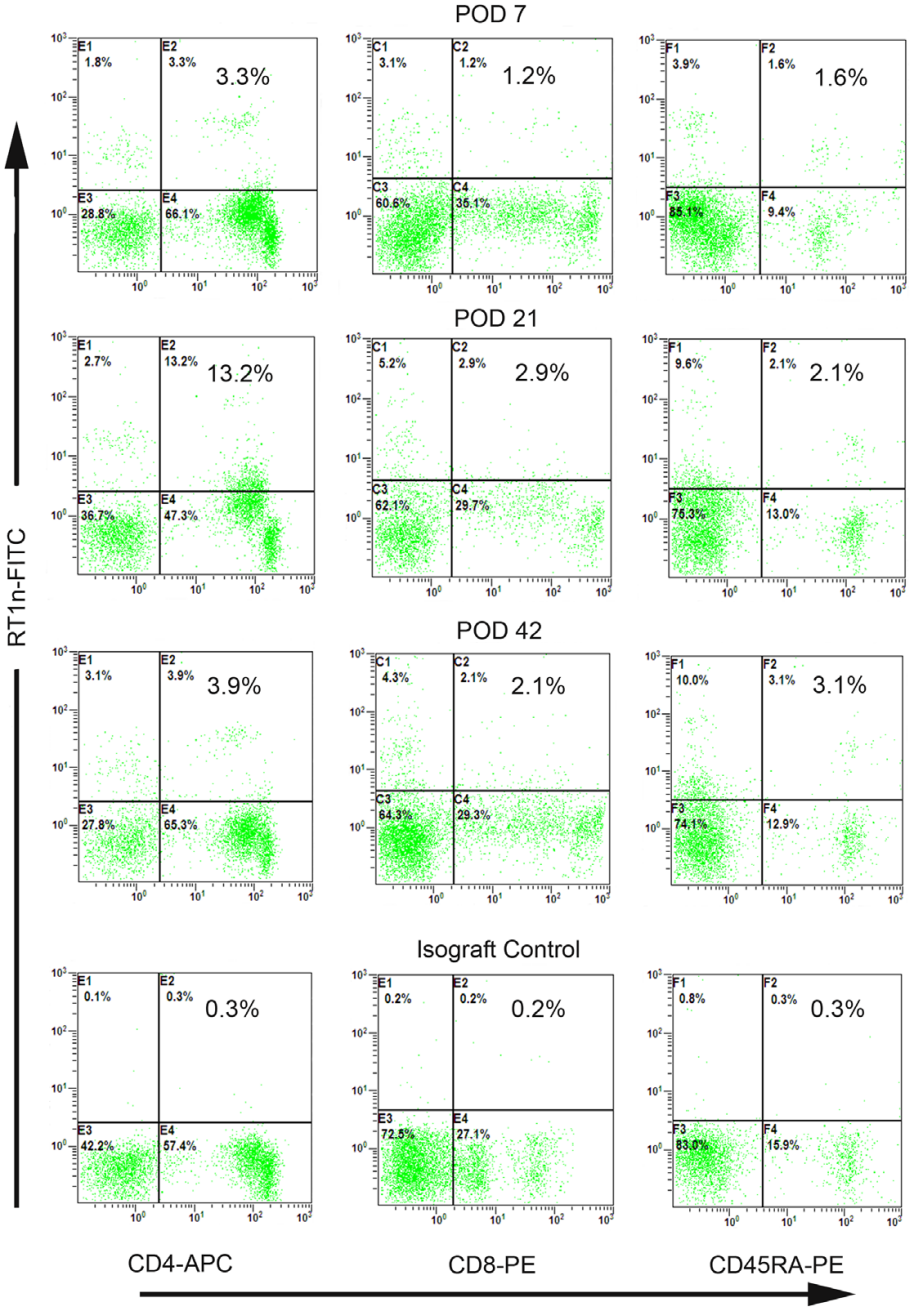
Dynamic changes in peripheral and myeloid chimerism. (a) Time kinetics of chimerism in groups 5 and 6. At POD 7, they showed almost the same chimerism level (*P*>0.05). At POD 21, the level of chimerism in group 6 was mildly decreased, but it increased sharply in group 5 (*P*<0.05). At POD 42, chimerism in group 5 returned to a total level 8.7%±0.6% but it decreased to background level in group 6 (*P*<0.05). In addition, donor-origin CD45RA+ B cells in group 5 prevailed beginning at POD 21 and maintained thereafter. (b) Myeloid chimerism was detectable at 6.3±0.5% at POD 100 in group 5 and it never appeared in group 6 when allografts were rejected (n = 6, *P*<0.05).

We quantified the donor-specific cells in the peripheral blood of LEW recipients in groups 3 and 4 by flow cytometry at POD 1 and 7. We quantified it in groups 5 and 6 at POD 7, 21, 42, and 100. The percentage of donor-origin cells in groups 3 and 4 peaked at POD 1 (7.4%±0.3% and 6.8%±0.9%, respectively). However, this chimerism declined swiftly. At POD 7, only 1.8%±0.8% and 1.6%±0.7% donor origin cells were detected in the peripheral blood in groups 3 and 4. In group 5, peripheral chimerism at POD 7 was assessed at 8%±0.9%, which was similar to the level observed in groups 3 and 4 at POD 1. A higher degree of chimerism was observed at POD 21 among 15.9%±1.6% of donor cells. Levels plateaued at 8.7%±0.6%. In group 6, the relative number of donor cells reached 7.5%±0.3%, 6.9%±0.6%, and 1.4%±0.3% at POD 7, 21, and 42, respectively. No differences in chimerism were observed at POD 7. At POD 21, the chimerism in group 5 peaked, but it showed moderate decreases in group 6. Differences between these two groups were found to be significant (*P*<0.05). At POD 42, chimerism in group 5 stabilized, but chimerism in group 6 decreased sharply down to nearly background levels. Differences between groups 5 and 6 were again found to be significant (*P*<0.05) (Fig. 3a). Dynamic changes in the peripheral chimerism of one representative recipient in group 5 are shown in Fig. 4.

### Myeloid chimerism in the bone compartments of recipients

**Figure 5:**
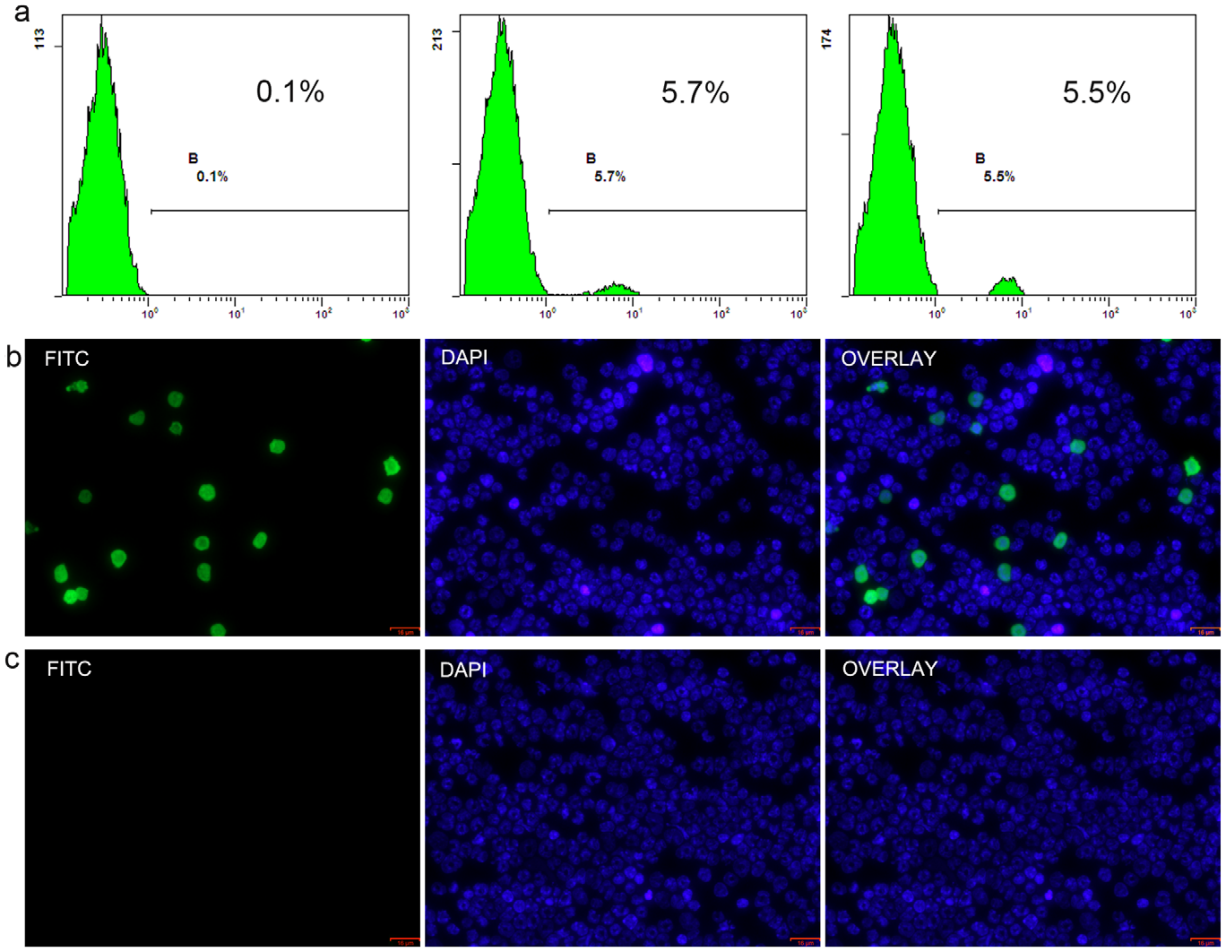
Flow cytometry analysis for myeloid chimerism and immunocytofluorescence staining for the presence of donor-origin cells in recipients' innate femurs. (a)The proportion of donor-origin cells observed from 2 representative recipients at POD 30 were at 5.7% and 5.5%, respectively, with the background level assessed at 0.1%. (b, c) The presence of RT1n cells in the contralateral femurs of recipients in group 5 and their absence in other groups was confirmed by immunocytofluorescence (×20).

Myeloid engraftment was a result of the homing of primitive donor-origin cells. This contributes to a constant chimerism in peripheral blood. Background noise analysis was performed in isograft recipients, which was not detected (N.D.). The proportion of RT1^n^ cells in the contralateral femurs of recipients in groups 3 and 4 on the day of rejection and in group 6 on POD 30 and on rejection day were also N.D (Fig. 5a-A). The proportion of donor-origin cells observed from 2 representative recipients at POD 30 and from the other 6 recipients at POD 100 were 5.7%, 5.5% (Figs. 5 a-B, C) and 6.3%±0.5%, respectively (Fig. 3b).

### Immunocytochemical staining confirmed myeloid engraftment

Donor-origin RT1^n^-FITC positive cells were detectable in the bone marrow cells of the contralateral innate femur from recipients in group 5 at POD 30 and 100. However, they did not appear in any other group. This confirmed that myeloid engraftment occurred solely in CsA-treated intact allogeneic hind limb recipients (Figs. 5b, 5c).

### Engraftment and tolerance to allografts accompanied by increased Foxp3^+^ Tregs with donor property

**Figure 6:**
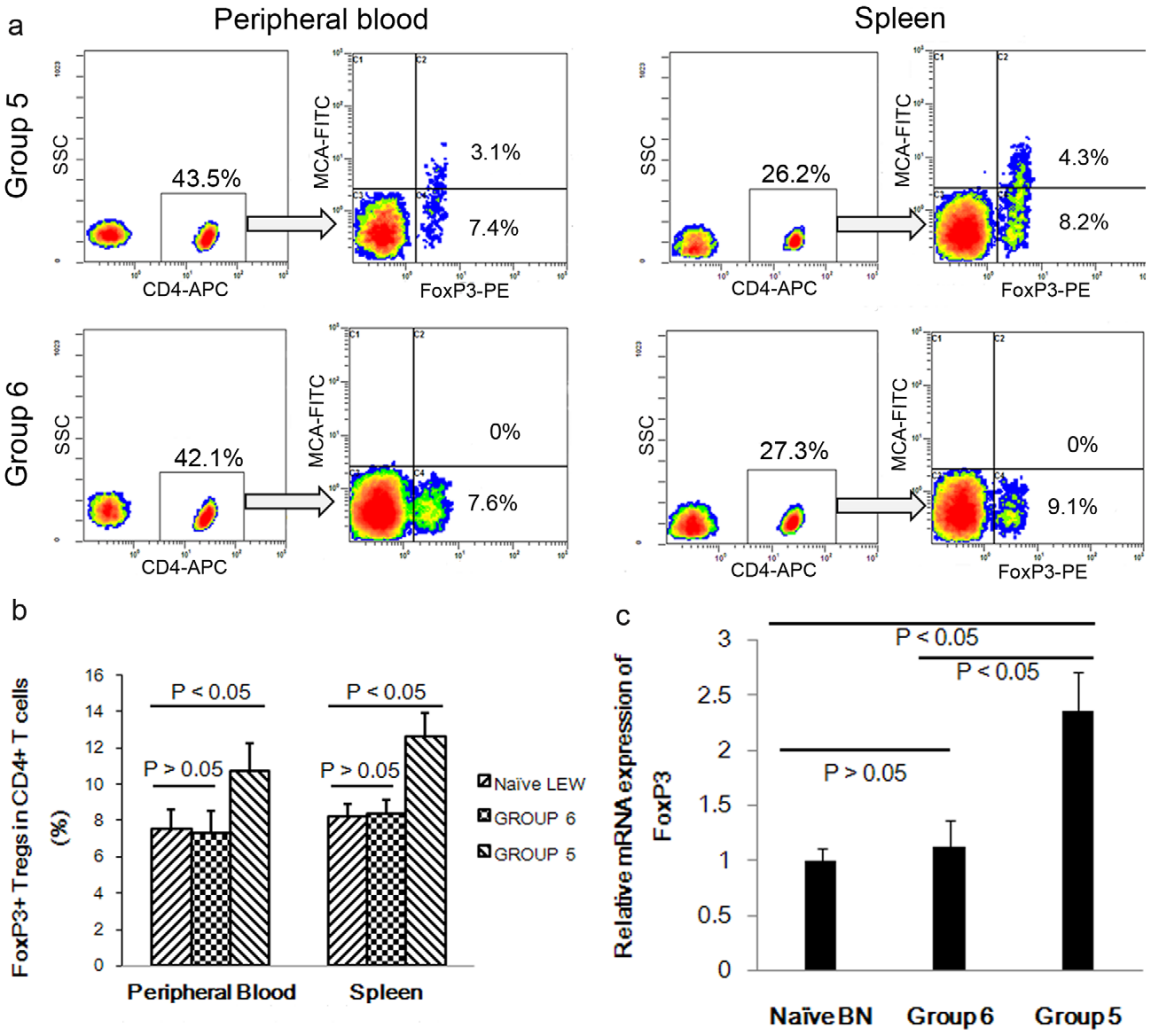
Location of FoxP3^+^ Tregs in rats receiving CsA treatment. (a) Representative dot plots of donor-specific FoxP3^+^ Tregs in peripheral blood and spleen from allograft recipients in groups 5 and 6 at the end of the observation period. Mononuclear cells were obtained by Ficoll solution centrifugation, followed by magnetic isolation of CD4+ T cells. Isolated CD4+ T cells were then co-stained with anti-rat mAb RT1^n^-FITC and intracellularly stained with anti-mouse/rat Foxp3-PE. All cells were analyzed by flow cytometry. The results demonstrated that donor-specific FoxP3^+^ Tregs were exclusively obtained in both the peripheral blood and spleens of long-term allograft recipients in group 5. (b) Further analysis indicated an average proportion of 10.7%±1.1% versus 7.6%±1.6% and 12.6%±0.7% versus 8.3%±1.1 FoxP3^+^ Tregs located in the peripheral blood and spleen in group 5 and 6, respectively, with significant differences (n = 6, *P*<0.05). (c) Expression of FoxP3 mRNA in the allografts was analyzed by real-time PCR normalized to GAPDH. Significantly higher levels of FoxP3 were observed in group 5 (n = 6, *P*<0.05).

To determine whether Foxp3^+^ Tregs contribute to the allograft survival in our model, we analyzed their locations in the peripheral blood and the spleen and the levels of mRNA expression in the graft of CsA-treated rats in groups 5 and 6 at the end of the observation period (i.e., at POD 100 or at the allograft rejection day). FoxP3^+^ Tregs in the peripheral blood were also analyzed at POD 30. These analyses showed that the frequency of Foxp3^+^ Tregs in the peripheral blood in groups 5 and 6 at POD 30 were both at the background levels obtained from the naïve LEW rats (data not shown). At POD 100, the proportion of Foxp3^+^ Tregs increased in both the peripheral blood and the spleens of recipient rats in group 5, with a high degree of FoxP3 mRNA expression in the allografts (Figs. 6b, 6c). However, when the grafts were rejected in group 6, Foxp3^+^ Tregs observed in either the peripheral blood or spleen, and Foxp3 mRNA expressions in the allografts were not significantly different from the background level. These increased levels of Foxp3^+^ Tregs in group 5 were further found to be donor-specific (Fig. 6a).

### Donor-specific Foxp3^+^ Tregs were required for donor hyporeactivity by one-way mixed lymphocyte proliferation assays

**Figure 7:**
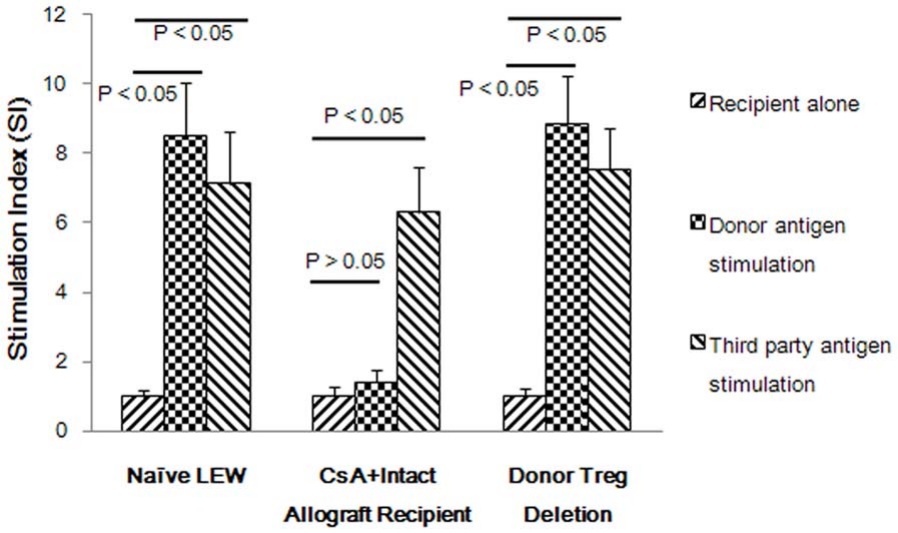
One-way MLR revealed a critical role of donor-specific FoxP3^+^ Tregs for the maintenance of tolerance. Baseline levels were assessed when naïve Lewis splenic cells were used as responders. Hyporeactivity to donor antigen stimulation and immunocompetence for third-party antigens stimulation (ACI rats) in recipients of group 5 at POD 100 was demonstrated. When donor-specific FoxP3^+^ Tregs were depleted from the responder populations, robust antidonor alloreactivity reappeared.

In an effort to elucidate the role of donor-specific FoxP3^+^ Tregs in the maintenance of tolerance, we performed *in vitro* one-way MLR assays at the end of the observation period. None of the splenic cells from recipient rats in group 5 showed any reactivity to donor antigen stimulation (S.I = 1.4±0.35), but they remained far more reactive to third-party antigen stimulation (S.I = 6.3±1.3) than baseline levels when naïve Lewis splenic cells were used as responders (S.I = 8.5±1.5 and 7.1±1.5 to donor and third-party antigens, respectively). However, when donor-specific FoxP3^+^ Tregs were removed from the responder populations, robust antidonor alloreactivity reappeared (S.I 8.8±1.4, roughly equal to the baseline level). Reactivity to the third-party antigens remained generally strong in these assays (Fig. 7). These *in vitro* data confirmed the donor-specific tolerance of recipients in group 5, which was consistent with the long-term graft survival observed in this group.

## Discussion

Conventional bone marrow cell transplantation has been found to be effective in inducing chimerism and tolerance, which eliminates the need for lifelong immunosuppression [19], [20]. However, this involves significant risk and a time-consuming process including recipient conditioning, donor bone marrow cell isolation, and the administration of substantial amounts of immunosuppressants for achieving transplanted cell engraftment and the repetitive detection for the presence of stable chimerism. In this way, it is not feasible for composite tissue allotransplantation, in which cadaveric donors are always used and recipients are not under the threat of death. Recently, it has been proposed that VBMT may be superior to bone marrow cell transplantation for the establishment of chimerism and the induction of tolerance. Several studies have suggested that femoral, sternal, mandibular elements within the allograft may serve as a substantial source of bone marrow cells, resulting in the development of donor-specific chimerism [11], [21], [22].

Rats make up the most frequent animal model of hind limb allotransplantation with vascularized bone marrow grafts. Previous studies have indicated that transplanted hind limb allografts containing bone marrow cells and stromal microenvironments facilitate allograft tolerance under certain immunosuppressive protocols, but the mechanism underlying this improved tolerance has not been extensively investigated. In this study, with a modified hind limb transplantation model, we observed that, under a minimally toxic recipient immunosuppressive protocol, the intact hind limb survived rejection-free for more than 100 days. However, when the bone components were removed, the allografts tended to be rejected within 2 months. Most importantly, we identified a critical role for donor-specific FoxP3^+^ Tregs in allografts survival.

Previous studies have shown that transplanted organs and CTAs contain large numbers of immunocompetent cells, including those of myeloid origin. These cells are called passenger leukocytes [23], [24]. Once vascular anastomoses are established, these leukocytes may migrate from grafted tissue to peripheral blood and colonize both lymphoid and non-lymphoid organs of the recipient. In this study, we observed that, shortly after operation, donor-origin RT1^n^ cells were detected in recipients in both the rejection and experimental groups. But due to the lack of immunosuppression, the number of RT1^n^ cells sharply declined during the course of the rejection process, regardless of whether the bone components had been removed. Likewise, with a tapered CsA protocol, the chimerism in B–R allograft recipients gradually declined and disappeared whenever the graft rejection process took less than 2 months. In contrast, under the same CsA treatment, the intact hind limb survived rejection-free for more than 100 days. Immunocytofluorescence analysis of bone marrow cells from the contralateral recipient innate femur at POD 30 showed the presence of donor-origin cells in intact allograft recipients and an absence of such cells from B–R allograft recipients. This indicated that the passenger leukocytes within the B–R allografts were unable to engraft to the bone marrow compartment of recipients under the tapered CsA protocol. The bone components were required for the induction of myeloid chimerism.

FoxP3^+^ Tregs have been shown to play a crucial role in maintaining allograft tolerance in experimental models of transplantation recently [25]. In order to disclose the mechanism underlying the long-term survival of transplants to rat recipients with myeloid chimerism, we investigated the contribution of FoxP3^+^ Tregs to allograft survival in our model. At POD 30, when myeloid chimerism has been successfully achieved in group 5, the frequency of Foxp3^+^ Tregs in the peripheral blood showed no differences from that of group 6. However, when at POD 100, donor-specific FoxP3^+^ Tregs were acquired only in intact hind limb recipient rats that had received CsA treatment. This treatment was therefore considered responsible for the donor-specific hyporeactivity by *in vitro* one-way MLR and may be required for the maintenance of tolerance to allografts as supported by the significantly higher FoxP3 mRNA expression in the long-term accepted allografts than in the rejected allografts. Our results are consistent with those of other, supporting the conclusion that Tregs is critical to the maintenance of allograft survival [26], [27].

These results conflict with published data, which state that long-term calcineurin inhibitors are associated with a decline of Tregs in patients receiving solid organ transplantation [28]. Considering the complex process of tolerance induction/maintenance to transplanted tissues and the relatively delayed appearance of donor-specific FoxP3^+^ Tregs in group 5, we deduce that the increased donor-specific FoxP3^+^ Tregs are likely to be induced by the myeloid chimerism established in intact allogeneic hind limb recipients under the minimally toxic CsA guarantee, which have immunoregulatory effects on the recipients and contribute to long-term allograft survival. However, this has yet to be confirmed. There are also data indicating that the effects of calcineurin inhibition on Treg function may be dose-dependent and that low doses may permit or even support Treg function [29].

We used a noninvasive laser Doppler flowmeter to monitor postoperative blood perfusion status. No significant differences were observed between any 2 groups that received the same treatment, indicating that the B–R procedure did not affect blood perfusion. Isotransplantation and rejection groups showed the same survival period, confirming that this procedure does not cause the final rejection of B–R allografts observed in the experimental groups.

In conclusion, using B–R hind limbs with all the same elements as the intact hind limbs except for its lack of bone components, we observed that, under a minimally toxic recipient immunosuppressive protocol, intact allogeneic hind limbs were able to survive rejection-free for more than 100 days. When the bone components were removed, early rejection episodes and graft loss were observed. Further investigation indicated that donor-specific FoxP3^+^ Tregs are required for donor hyporeactivity in *in vitro* one-way MLR and that they may be responsible for long-term allograft survival. The means by which these cells are generated merits further investigation. The B–R hind limb model might serve as a counterpart to tolerance induction studies of hind limb allotransplantation and so advance realization of the allotransplantation in clinical settings.

## Materials and Methods

### Animals

Inbred Brown-Norway [BN (RT1^n^)] rats and Lewis [LEW (RT1^1^)] rats with disparate major histocompatibility complexes (MHC) and minor histocompatibility antigens were purchased from Vital River, Inc. (Beijing, China). Eight-to-ten-week-old BN rats weighing 175–200 g were used as donors, LEW rats equal in age and body weight were used as recipients. The research protocol was approved by the Experimental Animal Committee of the Fourth Military Medical University. All rats received humane care in compliance with the *Guide for the Care and Use of Laboratory Animals* published by the National Institutes of Health.

The experimental design is described in Table 1. Forty-eight hind limb transplants were performed. Isograft transplantations were performed in groups 1 and 2 between genetically identical LEW rats (Isotransplantation Groups). Allograft transplantations were performed between BN rats and LEW rats in groups 3 through 6. The rats in groups 3 and 4 (Rejection Groups) were given 1 mL. In groups 5 and 6 (Experimental Groups), all recipients were treated with tapered CsA, which was administered on day 0 at a standard dose of 16 mg/kg per day for 1 week, tapered to 2 mg/kg per day over 4 weeks, and maintained at this level thereafter. The femur, tibia, and fibula were kept intact in groups 1, 3, and 5 (intact grafts), but they were removed in groups 2, 4, and 6 (bone-removal grafts, B–R grafts) before transplantation.

**Table 1:** Experimental design and survival time of the transplanted grafts.

Groups	Surgical procedure	Treatment	Survival time(d)
Group1(n = 8)	Isotransplantation	No	>100
Group2(n = 8)	Isotransplantation(b–r)	No	>100
Group3(n = 8)	Allotransplantation	N.S	8,9,8,11,10,9,10,12
Group4(n = 8)	Allotransplantation(b–r)	N.S	9,8,11,12,11,10,8,12
Group5(n = 8)	Allotransplantation	CsA	30#,30#, 100*,100*,100*,100*,100*,100*
Group6(n = 8)	Allotransplantation(b–r)	CsA	30#,30#,46,49,47,49,48,51

# At POD 30, 2 recipients in groups 5 and 6 were killed for myeloid chimerism evaluation.

* At POD 100, the other 6 recipients in group 5 were killed for evaluation of myeloid chimerism and determination of underlying mechanisms.

### Surgical procedure

#### Bone-removal hind limb preparation

After general anesthesia with sodium pentobarbital (50 mg/kg), the donor BN rats were shaved and below the inguinal level and their skin was sterilized with 75% ethanol solution. A longitudinal incision was made from the sacroiliac joint through the anterior aspect of the ankle joint and the knee joint along the lateral femoral intermuscular septum of the recipient's right hind limb. The femur, tibia, and fibula were removed after the dissection of all adherent tissues and ligation of the vascular branches supplying the femur and tibia. Then a 1.5 mm Kirschner wire was put in place of the femur and tibia. The incisions were then closed with 3/0 sutures. After preparation, B–R hind limbs were immediately transplanted to recipient rats.

#### Transplantation

Hind limb transplantation was performed according to the technique described by Doi [30]. Briefly, a circumferential skin incision was made in the recipients rear middle thighs. Then the femoral artery, vein, and nerve were dissected, clamped, and transected proximal to the superficial epigastric artery. The limb was amputated at the mid-femoral level. The donor limb was prepared in the same way. Donor limbs were then attached to the recipient's femoral stump with 1.5 mm Kirschner wire. After femoral vessels of the donor and recipient were anastomosed and ascertained to be patent, the incision was closed. Three milliliters of N.S. was injected intraperitoneally to compensate for perioperative fluid loss.

### Evaluation of graft survival and assessment of graft-versus-host disease

After the operation, clinical manifestations of rejection, such as changes in color and temperature, progressive edema, loss of hair, desquamation, ulceration, epidermolysis, exudation, stiffness, and progressive shrinkage were recorded and evaluated daily. Clinical criteria for the appearance of graft-versus-host disease (GVHD) included diffuse erythema, hyperkeratosis of the foot pads, dermatitis, weight loss, and diarrhea.

### Quantitation of blood perfusion

All recipients were anesthetized under 1% isoflurane in 100% oxygen flow at 1 L/min on postoperative days (POD) 1, 4, and 7. The blood perfusion of both hind limbs was determined by Laser Doppler Flowmeter (PeriFlux System 5000, Perimed, Stockholm, Sweden) scanning, which generated computerized pseudo-color images. To minimize variation, all animals were placed in a supine position on a 37°C heating pad during scanning. After the images were recorded, the perfusion value, indicated by color, was defined within a range of 0 to 1000. Blood perfusion of the hind limb grafts was expressed as the ratio of perfusion value of the transplanted hind limb to that of the contralateral innate hind limb.

### Histopathology

Tissue sections were cut from 6 µg skin samples of the grafts for hematoxylin/eosin staining. Each tissue section was evaluated by a pathologist and the histopathological grade of each tissue sample was determined according to previously published criteria [31].

### Chimerism evaluation by flow cytometry analysis

One hundred microliters of peripheral blood was collected from recipients in all groups. Bone marrow was harvested from the contralateral innate femurs of 2 representative recipients at POD 30 and from the other 6 animals either at POD 100 or when the allografts in all allotransplantation groups were rejected. Peripheral blood samples were incubated with the combination of mouse anti-rat mAb RT1^n^-FITC (BN MHC Class I, clone MCA 156/OX-27, Serotec, U.K.) and either mouse anti-rat mAb of T-cell CD4-APC (clone W3/25), CD8a-PE (clone OX-8), and B-cell CD45RA-PE (clone OX-33; Biolegend, San Diego, CA, U.S.) for 30 minutes at 37°C away from light. Then red cells were lysed by the addition of lysing buffer (FACS lysing solution; Bio Legend Co.) Cells were incubated for 10 min at room temperature. Cells were finally suspended in 1% paraformadehyde for 30 min and subjected to an EPICS XL flow cytometry (Beckman-Coulter, Fullerton, CA, U.S.). Bone marrow cells from the contralateral innate femurs were loaded on the 1.077 g/mL Ficoll density gradient solution and centrifuged at 316 g for 20 min. The white membrane layer was resuspended in PBS. One million cells stained with mouse anti-rat mAb RT1^n^-FITC were quantified by flow cytometry and analyzed with Expo 32 ADC analysis software (Beckman-Coulter, Fullerton, CA, U.S.).

### Immunocytochemical staining

Bone marrow cells from the contralateral innate femurs stained with mouse anti-rat mAb RT1^n^-FITC were further stained with DAPI for 5 min. They were then washed with PBS and smeared on the microscope slide, and examined for the presence of donor cells by the Olympus FSX100 Bio Imaging Navigator (OLYMPUS, Japan).

### Detection of donor-specific FoxP3^+^ Tregs by flow cytometry analysis

One and a half milliliters of peripheral blood and 1/3 single spleen cell suspensions from recipients in the experimental groups were isolated at the end of the observation period through 1.077 g/mL Ficoll density gradient. Then >95% pure CD4^+^ T cells were magnetically isolated using a CD4^+^ T cell isolation kit in accordance with the manufacturer's protocol (Miltenyi Biotec, Bergisch Gladbach, Germany). Isolated CD4^+^ T cells were then stained with mouse anti-rat mAb RT1^n^-FITC for 30 min at 4°C. The stained cells were washed, fixed, and permeabilized with saponin 20% (Sigma-Aldrich) for 30 min at 37°C. Permeabilized cells were stained with Anti-Mouse/Rat Foxp3 PE (Cat. 12577382; eBioscience). All cells were quantified by flow cytometry and analyzed with Expo 32 ADC analysis software.

### RNA isolation and real-time PCR

Total RNA from allografts of groups 5 and 6 were isolated with Trizol Reagent (Invitrogen, San Diego, CA, U.S.) according to the manufacturer's instruction. Five hundred ng of RNA was reverse-transcribed using PrimeScript RT Kit (TaKaRa, Dalian, China) in a final volume of 10 µL. Real-time PCR was performed in the Bio-Rad IQ^5^ Real-Time System (Bio-Rad, Hercules, CA, U.S.) by using a SYBR® Premix Ex Taq™ II kit (TaKaRa, Dalian, China) in a 20 µL volume of the PCR reaction solution. Primer sequences were as follows: FoxP3: forward 5′-CTTCAGACAGCTTGTTTGCTGTG-3′, reverse 5′-GGGCCGCATATTATGGTAC-TTG-3′; GAPDH: forward 5′-GGCACAGTCAAGGCTGAGAATG-3′, reverse 5′- ATGGTGGTGAAGACGCCAGTA-3′ The cycling conditions used were as follows: initial denaturation at 95°C for 30 s, followed by 40 cycles with denaturation at 95°C for 10 s, annealing at 60°C for 10 s, and elongation at 72°C for 15 s. The expression level of GAPDH was used for internal control.

### *In vitro* one-way mixed lymphocyte reaction assay

One-way mixed lymphocyte reaction (MLR) assay was used to determine donor-specific tolerance. Spleens were harvested and mashed to yield single cell suspensions. After red cell clearance, they were adjusted to 2×10^6^/mL in RPMI 1640 medium (Gibco, U.S.) containing 10% FBS. Responder cells were isolated from naïve Lewis rats or recipients in group 5 at POD 100, when they were killed. In order to determine the role of FoxP3^+^ Tregs, parts of the responder cells from recipient rats in group 5 were donor-specific FoxP3^+^ Tregs magnetically depleted. Stimulator cells were isolated from the spleens of BN and third-party August-Copenhagen-Irish (ACI) rats and inactivated by Mitomycin C (Sigma, St. Louis, MO, U.S.) at 50 µg/mL, 37°C for 30 min preincubation. Two-hundred thousand responder cells and equal numbers of stimulator cells were then cocultured in 200 µL of RPMI 1640 medium containing 10% FBS in 96-well plates in a humidified 37°C, 5% CO2 incubator. After 3 days of incubation, cultures were pulsed with 1 µCi [^3^H] Thymidine for 18 h and assessed by a β-scintillation counter. The stimulation index (SI) was determined by assessing the ratio of the counts per minute (c.p.m.) generated in response to each stimulator to that of the culture medium alone (in the absence of stimulators).

### Statistical analysis

All data were expressed as mean ± SEM. To compare average perfusion between B–R hind limbs and intact hind limbs subjected to the same treatment or when average chimerism and FoxP3^+^ Tregs as well as FoxP3 mRNA expression between groups 5 and 6 were compared, an independent sample T-test was performed. All statistical analyses were performed using SPSS 13.0. Statistical differences were considered significant at *P*<0.05.

## References

[1] Ziegler-Graham K, MacKenzie EJ, Ephraim PL, Travison TG, Brookmeyer R (2008) Estimating the prevalence of limb loss in the United States: 2005 to 2050. Arch Phys Med Rehabil 89: 422–429. 18295618 10.1016/j.apmr.2007.11.005

[2] Robinson E, Rumsey N, Partridge J (1996) An evaluation of the impact of social interaction skills training for facially disfigured people. Br J Plast Surg 49: 281–289. 8774241 10.1016/s0007-1226(96)90156-3

[3] Levine E, Degutis L, Pruzinsky T, Shin J, Persing JA (2005) Quality of life and facial trauma: psychological and body image effects. Ann Plast Surg 54: 502–510. 15838211 10.1097/01.sap.0000155282.48465.94

[4] Ye EM (1998) Psychological morbidity in patients with facial and neck burns. Burns 24: 646–648. 9882064 10.1016/s0305-4179(98)00081-3

[5] Ravindra KV, Wu S, Bozulic L, Xu H, Breidenbach WC, et al (2008) Composite tissue transplantation: a rapidly advancing field. Transplant Proc 40: 1237–1248. 18589081 10.1016/j.transproceed.2008.04.003 PMC2692668

[6] Rossini AA, Dale GL, Mordes JP (1999) Induction of immunologic tolerance for transplantation. Physiol Rev 79: 99–141. 9922369 10.1152/physrev.1999.79.1.99

[7] Rifle G, Mousson C (2003) Donor-derived hematopoietic cells in organ transplantation: a major step toward allograft tolerance?. Transplantation 75 (9Suppl) 3S–7S. 12819482 10.1097/01.TP.0000067943.90241.73

[8] Foster RD, Pham S, Li S, Aitouche A (2003) Long-term acceptance of composite tissue allografts through mixed chimerism and CD28 blockade. Transplantation 76: 988–994. 14508367 10.1097/01.TP.0000079827.91675.A3

[9] Hettiaratchy S, Randolph MA, Petit F, Lee WP, Butler PE (2004) Composite tissue allotransplantation–a new era in plastic surgery?. Br J Plast Surg 57: 381–391. 15191817 10.1016/j.bjps.2004.02.012

[10] Arsian E, Klimczak A, Siemionow M (2007) Chimerism induction in vascularized bone marrow transplants augmented with bone marrow cells. Microsurgery 27: 190–199. 17326192 10.1002/micr.20330

[11] Kulahci Y, Klimczak A, Madajka M, Altuntas S, Siemionow M (2010) Long-term survival of composite hemiface/mandible/tongue allografts correlates with multilineage chimerism development in the lymphoid and myeloid compartments of recipients. Transplantation 90: 843–852. 20697324 10.1097/TP.0b013e3181f28bb0

[12] Hori S, Nomura T, Sakaguchi S (2003) Control of regulatory T cell development by the transcription factor Foxp3. Science 299: 1057–1061. 12522256 10.1126/science.1079490

[13] Tang Q, Bluestone JA, Kang SM (2012) CD4^+^ FoxP3^+^ regulatory T cell therapy in transplantation. Journal of Molecular Cell Biology 4: 11–21. 22170955 10.1093/jmcb/mjr047 PMC3695644

[14] Bonde S, Chan KM, Zavazava N (2008) ES-Cell Derived Hematopoietic Cells Induce Transplantation Tolerance. PLoS ONE 3 (9) e3212. 18791641 10.1371/journal.pone.0003212 PMC2527660

[15] Stevens HP, Hovius SE, Heeney JL, van Nierop PW, Jonker M (1991) Immunologic aspects and complications of composite tissue allografting for upper extremity reconstruction: a study in the rhesus monkey. Transplant Proc 23: 623–625. 1990628

[16] Lanzetta M, Petruzzo P, Margreiter R, Dubernard JM, Schuind F, et al (2005) The International Registry on Hand and Composite Tissue Transplantation. Transplantation 79: 1210–1214. 15880072 10.1097/01.tp.0000157118.28394.fa

[17] Piza-Katzer H, Ninkovic M, Pechlaner S, Gabl M, Ninkovic M, et al (2002) Double hand transplantation: functional outcome after 18 months. J Hand Surg Br 27: 385–390. 12162985 10.1054/jhsb.2002.0759

[18] Dubernard JM, Petruzzo P, Lanzetta M, Parmentier H, Martin X, et al (2003) Functional results of the first human double-hand transplantation. Ann Surg 238: 128–136. 12832975 10.1097/01.SLA.0000078945.70869.82 PMC1422660

[19] Brouha PC, Iidstad ST (2001) Mixed allogeneic chimerism. Past, present, and prospects for the future. Transplantation 72 (Suppl 8) S36–S42. 11888155

[20] Siemionow M, Oke R, Ozer K, Izycki D, Prajapati R (2002) Induction of donor-specific tolerance in rat hind-limb allografts under antilymphocyte serum and cyclosporine A protocol. J Hand Surg Am 27: 1095–1103. 12457363 10.1053/jhsu.2002.36524

[21] Tai CY, France MA, Strande LF, Eydelman R, Sheng X, et al (2003) An extraperitoneal isolated vascularized bone marrow transplant model in the rat. Transplantation 75: 1591–1593. 12792520 10.1097/01.TP.0000061490.85273.29

[22] Santiago SF, de Faria W, Khan TF, Gandia CE, Misiakos EP, et al (1999) Heterotopic sternum transplant in rats: A new model of a vascularized bone marrow transplantation. Microsurgery 19: 330–334. 10586198 10.1002/(sici)1098-2752(1999)19:7<330::aid-micr8>3.0.co;2-i

[23] Kreisel D, Petrowsky H, Krasinskas AM, Krupnick AS, Szeto WY, et al (2002) The role of passenger leukocyte genotype in rejection and acceptance of rat liver allografts. Transplantation 73: 1501–1507. 12023631 10.1097/00007890-200205150-00022

[24] Starzl TE, Zinkernagel RM (1998) Antigen localization and migration in immunity and tolerance. N Engl J Med 339: 1905–1913. 9862947 10.1056/NEJM199812243392607 PMC2963936

[25] Denecke C, Bedi DS, Ge X, Kim IK, Jurisch A, et al (2010) Prolonged Graft Survival in Older Recipient Mice Is Determined by Impaired Effector T-Cell but Intact Regulatory T-Cell Responses. PLoS ONE 5 (2) e9232. 20169060 10.1371/journal.pone.0009232 PMC2821908

[26] Eljaafari A, Badet L, Kanitakis J, Ferrand C, Farre A, et al (2006) Isolation of regulatory T cells in the skin of a human hand-allograft, up to six years posttransplantation. Transplantation 82: 1764. 17198273 10.1097/01.tp.0000250937.46187.ca

[27] Bozulic LD, Wen Y, Xu H, Ildstad ST (2011) Evidence that FoxP3+ regulatory T cells may play a role in promoting long-term acceptance of composite tissue allotransplants. Transplantation 91: 908–915. 21304439 10.1097/TP.0b013e31820fafb4 PMC3592205

[28] Demirkiran A, Hendrikx TK, Baan CC, van der Laan LJ (2008) Impact of immunosuppressive drugs on CD4+CD25+FOXP3+ regulatory T cells: does in vitro evidence translate to the clinical setting?. Transplantation 85: 783–789. 18360256 10.1097/TP.0b013e318166910b

[29] Brandt C, Pavlovic V, Radbruch A, Worm M, Baumgrass R (2009) Low-dose cyclosporine A therapy increases the regulatory T cell population in patients with atopic dermatitis. Allergy 64: 1588–1596. 19432936 10.1111/j.1398-9995.2009.02054.x

[30] Doi K (1979) Homotransplantation of limbs in rats. Plast Reconstr Surg 64: 613–621. 504483

[31] Cendales LC, Kirk AD, Moresi JM, Ruiz P, Kleiner DE (2006) Composite tissue allotransplantation: classification of clinical acute skin rejection. Transplantation 81: 418–422. 16477229 10.1097/01.tp.0000185304.49987.d8

